# Reactive oxygen and nitrogen species in sepsis-induced hepatic microvascular dysfunction

**DOI:** 10.1007/s00011-012-0562-3

**Published:** 2012-10-18

**Authors:** Georg Singer, Karen Y. Stokes, D. Neil Granger

**Affiliations:** 1 Department of Pediatric Surgery, Medical University of Graz, Graz, Austria; 2 Department of Molecular and Cellular Physiology, Louisiana State University Health Sciences Center, 1501 Kings Highway, Shreveport, LA 71130-3932 USA

**Keywords:** Nitric oxide, NADPH oxidase, iNOS, eNOS, nNOS, SOD

## Abstract

**Objective and design:**

Hepatic microvascular dysfunction is a critical event in the development of liver failure during sepsis. Activated blood cells and reactive oxygen and nitrogen species (RONS) have been implicated in the pathogenesis of sepsis.

**Methods:**

Intravital-videomicroscopy was used to determine whether RONS contribute to the recruitment of leukocytes/platelets in the hepatic microvasculature during sepsis. Six hours following cecal-ligation and puncture (CLP), disturbances of the hepatic microvasculature were assessed in WT-mice (C57Bl/6 J; *n* = 8), in mice lacking gp91^phox^(*n* = 5), overexpressing superoxide-dismutase (SOD, *n* = 8), in WT-mice treated with a NOS-inhibitor (l-NAME, *n* = 5), lacking nNOS, eNOS or iNOS (*n* = 5 each), treated with the NO-donor DetaNO (*n* = 5), in WT-mice treated with gadolinium-chloride (GdCl_2_, *n* = 5) and compared to a group of WT-mice following a sham operation (*n* = 8). Six hours post-CLP, the adhesion of leukocytes and platelets in terminal hepatic venules (THV) and sinusoids was quantified.

**Results:**

In WT-mice, CLP elicited increases in the number of adherent leukocytes and platelets. Similar responses to CLP were noted in mice overexpressing SOD or lacking either eNOS or gp91^phox^. The blood-cell recruitment was significantly blunted in septic iNOS-knockout mice and this response was reversed by pre-treatment with DetaNO.

**Conclusion:**

These findings suggest that iNOS-derived NO is a determinant of the pro-inflammatory phenotype assumed by the hepatic microvasculature during sepsis.

## Introduction

Despite recent advances in intensive care medicine, sepsis remains a serious medical problem, with an incidence of about three cases per 1,000 population and an estimated annual cost of $16.7 billion in the US alone [1]. In septic patients, the incidence of secondary organ dysfunction is about 30 %, resulting in a mortality rate of 50–80 %, depending on the number of organs involved [2]. Because of its primary role in metabolism and host defense and its contribution to the production of inflammatory mediators and coagulation factors, the liver is considered to play a major role in the initiation of the multiple organ dysfunction during sepsis [3]. Microcirculatory failure resulting from activation and recruitment of leukocytes in the hepatic microvasculature is thought to be a crucial event in the development of liver dysfunction [4]. The recruitment of adherent leukocytes in the liver is accompanied by impaired sinusoidal perfusion [5]. Although neutrophils contribute to defense against bacteria in sepsis, overwhelming activation of these phagocytic cells can elicit tissue damage [6]. There is also growing evidence for involvement of other blood cell populations, such as platelets, in the development of hepatic microvascular dysfunction following septic stimuli [7].

The production of reactive oxygen species (ROS) by immune cells has been attributed to NADPH oxidase, which consists of six subunits including gp91^phox^ (Nox2) [8]. NADPH oxidase transfers reducing equivalents from NADPH to oxygen, resulting in the generation of superoxide [9]. Endogenous antioxidant enzymes, such as superoxide dismutase (SOD) help protect tissues by catalyzing the dismutation of superoxide to oxygen and hydrogen peroxide [10]. Mutations of the NADPH oxidase subunits result in polymorphonuclear neutrophils that are incapable of the respiratory burst and have diminished bactericidal function, highlighting the integral role of NADPH oxidase during sepsis [11]. However, the contribution of NAPDH oxidase and ROS to the hepatic microvascular dysfunction in sepsis remains poorly understood. Therefore, one of the major objectives of this study was to assess the contribution of NADPH oxidase-derived superoxide to the microvascular response to sepsis. We hypothesized that both deletion of gp91^phox^ and overexpression of SOD would result in an attenuation of the microvascular disturbances caused by polymicrobial sepsis.

Nitric oxide (NO) has also received considerable attention as a mediator of tissue responses to sepsis. Three different isoforms of NO synthases (NOS) have been described: neuronal NOS (nNOS), endothelial NOS (eNOS) and inducible NOS (iNOS) [12]. In addition to its role as an endogenous vasodilator, NO (or derivatives thereof) has also been implicated as a mediator of cytotoxicity and inflammation [13]. Consequently, a variety of nonselective and selective inhibitors of the different NOS isoforms have been used to assess the contribution of NO to sepsis. A second major objective of this study was to determine whether mice lacking either iNOS, eNOS or nNOS exhibit an altered leukocyte and/or platelet recruitment response in mice subjected to sepsis induced by cecal ligation and puncture (CLP). We hypothesized that deletion of the different isoforms of NOS selectively alters the microvascular disturbances observed during sepsis.

## Materials and methods

### Animals

For the experiments, male C57Bl/6 J (WT), C57BL/6-Tg(SOD1)3Cje/J (SOD-tg), B6.129S6-Cybbtm1Din/J (gp91^phox^-ko), B6.129P2-Nos3tm1Unc/J (eNOS-ko), B6.129P2-Nos2tm1Lau/J (iNOS-ko), B6129SF2/J (nNOS-controls) and B6;129S4-Nos1tm1Plh/J (nNOS-ko) mice (6 weeks old; *n* = 5–8 per group) were purchased from Jackson Laboratory (Bar Harbor, ME). All mice except the nNOS-ko were on a C57Bl/6 background. Animal handling procedures were approved by the LSU Health Sciences Center Institutional Animal Care and Use Committee and were in accordance with the guidelines of the American Physiological Society.

### Experimental protocols

First, the hepatic microvascular responses at 6 h following CLP were assessed in SOD-tg and gp91^phox^-ko mice and compared to wild type (WT) mice at 6 h following a sham operation or a CLP procedure. Mean arterial pressure (MAP), serum alanine aminotransferase (ALT) levels (a serum marker of hepatocellular injury) and blood leukocyte and platelet counts were obtained in each of these experiments.

Additionally, the hepatic microvascular responses 6 h post-CLP were determined in a group of WT mice treated with the nonspecific NOS inhibitor n-nitro-l-arginine methyl ester (l-NAME; 1 g/L; Bachem Americas Inc, CA) in drinking water for 7 days and compared to eNOS-ko, nNOS-controls, nNOS-ko and iNOS-ko mice. The l-NAME administered via drinking water has been repeatedly used for inhibiting NOS activity [14, 15]. Another group of iNOS-ko mice received an intraperitoneal injection of 50 μg/kg body weight diethylenetriamine NONOate (DETA-NO; Cayman Chemical) in normal saline immediately after the induction of sepsis [16]. Another group of WT mice received an intravenous injection of 20 mg/kg gadolinium chloride (GdCl_3_) 48 h prior to induction of sepsis to deplete Kupffer cells (WT-GdCl_3_-CLP) [17]. The results were compared to WT mice 6 h following either a sham or a CLP procedure. The MAP, blood cell counts, and serum ALT were also measured in these groups.

Blood was collected following CLP in different groups (WT-sham, WT-CLP, eNOS-ko + CLP, iNOS-ko + CLP, iNOS-ko + CLP + DetaNO, WT-GdCl_3_ + CLP and WT-GdCl_3_-sham) to determine serum concentrations of a panel of cytokines [tumor necrosis factor alpha (TNF-α), interleukin 6 (IL-6), monocyte chemoattractant protein—1 (MCP-1), interleukin 10 (IL-10), interferon gamma (IFN-γ), and interleukin 12 (IL-12)].

### Cecal ligation and puncture (CLP)

To induce sepsis, the CLP procedure was used as described previously [18]. Briefly, animals were anesthetized with ketamine hydrochloride (150 mg/kg i.m.) and xylazine (7.5 mg/kg i.m.). A midline laparotomy was performed, the cecum was exteriorized and ligated distal to the ileocecal valve, without causing intestinal obstruction. The cecum was perforated three times using a 20-gauge needle (top, middle and bottom third) and squeezed gently to extrude fecal contents that were spread around the cecum using a cotton swab. The incision was closed using two layers of sutures. Each mouse received 1 ml of normal saline subcutaneously for fluid resuscitation. The animals were allowed free access to standard chow and water after induction of sepsis. In sham animals, the cecum was exteriorized without ligation and puncture. All other procedures were identical to the CLP groups.

### Isolation of platelets

Platelets were obtained from the blood of corresponding donor mice and labeled with the fluorchrome carboxyfluorescein diacetate succinimudyl ester (CFDASE; Molecular Probes, Eugene, OR) as described previously [18]. This isolation process does not cause activation of platelets as determined by P-selectin expression [19].

### Intravital microscopy

The animals were re-anesthetized 6 h after the induction of CLP. The right jugular vein, and left carotid artery (for measurement of MAP) were cannulated. The abdomen was opened, the mice were placed on their left side and the left liver lobe was laid on a Plexiglas microscope stage and covered with a piece of gauze moistened with saline to avoid dehydration. In order to label leukocytes, 120 μl of rhodamine 6G was infused into the jugular catheter and allowed to circulate for 5 min. This was followed by i.v. infusion of labeled platelets (100 × 10^6^) over a period of 5 min, which were allowed to circulate for an additional 5 min before the observation period. This yields about 10 % of the total murine platelet count. The hepatic microvasculature was visualized using an inverted microscope (Nikon Diaphot) equipped with a 75 W XBO lamp with a 40× objective lens (40/0.65; Nikon). Microscopic images were received by a fluorescent camera (C 2400, Hamamatsu) and projected onto a monitor (PVM-2030, Sony Trinitron). For offline evaluation, the images were recorded on a VHS video recorder (EWV 404, Emerson). The liver surface was scanned for 3–5 venules, each of which was recorded for 1 min.

One hundred micron segments of terminal hepatic venules (THV) with diameters ranging from 15 to 45 μm were observed. Leukocytes and platelets were classified according to their interaction with the vessel wall. Cells were considered as firmly adherent cells if they remained stationary on the vessel wall for more than 10 s and were quantified as the number per mm^2^ venular wall (calculated assuming cylindrical vessel geometry) [20]. Within sinusoids, blood cells were considered stationary if they did not move for the entire observation period of 1 min and were expressed as numbers per mm^2^ liver surface. Sinusoidal perfusion failure was quantified by the number of nonperfused sinusoids (given as a ratio between nonperfused and total sinusoids per field of view). A sinusoid was considered nonperfused if no white blood cells were observed flowing through it.

### Blood cell counts

Leukocyte and platelet counts were performed with the aid of a hemocytometer using blood derived from the arterial catheter at the end of each experiment. The blood samples (25 μl) were mixed with 465 μl 3 % acetic acid and 10 μl 1 % crystal violet.

### Alanine aminotransferase (ALT) activity

At the end of the experiment, blood was collected from the arterial catheter and immediately centrifuged at 8,000*g* for 5 min. Serum was collected and frozen at −80 °C. ALT was measured spectrophotometrically using a kit from ThermoDMA (Louisville, CO). Data are presented as units per liter at 37 °C.

### Serum concentration of cytokines

Separate groups of mice underwent sham operation or CLP, but did not receive fluorescent dyes or cells. Heparinized blood was drawn from a catheter placed in the carotid artery to obtain serum for cytokine measurements. A cytometric bead array was used to measure the concentrations of TNF-α, IL-10, MCP-1, IL-6, IL-12 and IFN-γ in the serum samples as per the kit instructions (BD Biosciences, CA). The samples were analyzed on a FACS Caliber. The serum cytokine levels are expressed as pg/ml serum.

### Statistical analysis

All values are reported as mean ± SEM. The ANOVA with Scheffe’s post hoc test was used for statistical comparison of experimental groups. A *P* value of <0.05 was considered statistically significant.

## Results

### Reactive oxygen species experiments

The data for MAP, serum ALT levels, and blood leukocyte and platelet counts in WT, SOD-tg and gp91^phox^-ko mice following CLP are presented in Table 1. While the MAP and ALT levels were not affected by CLP in the tested strains of mice, CLP caused a significant decrease in both leukocyte and platelet counts in WT, SOD-tg and gp91^phox^-ko mice.

**Table 1:** Mean arterial pressure (MAP), serum ALT concentration, and blood leukocyte and platelet counts in WT mice and in mutant mice targeting reactive oxygen species

	MAP (mmHg)	ALT (IU/l)	Leukocyte count (#/μl)	Platelet count (# × 10^3^/μl)
WT-sham (*n* = 4)	60 ± 3	45 ± 9	4380 ± 250	1132 ± 33
WT + CLP (*n* = 7)	51 ± 4	53 ± 7	980 ± 150^*^	544 ± 34^*^
SOD-tg + CLP (*n* = 5)	68 ± 7	52 ± 4	2330 ± 440^*^	831 ± 57^*^
gp91^phox^-ko + CLP (*n* = 5)	59 ± 7	49 ± 5	2220 ± 340^*^	688 ± 76^*^

Values are mean ± SE

*MAP* mean arterial pressure, *ALT* alanine aminotransferase, *SOD* superoxide dismutase, *CLP* cecal ligation and puncture

* *p* < 0.05 versus WT sham

**Fig. 1:**
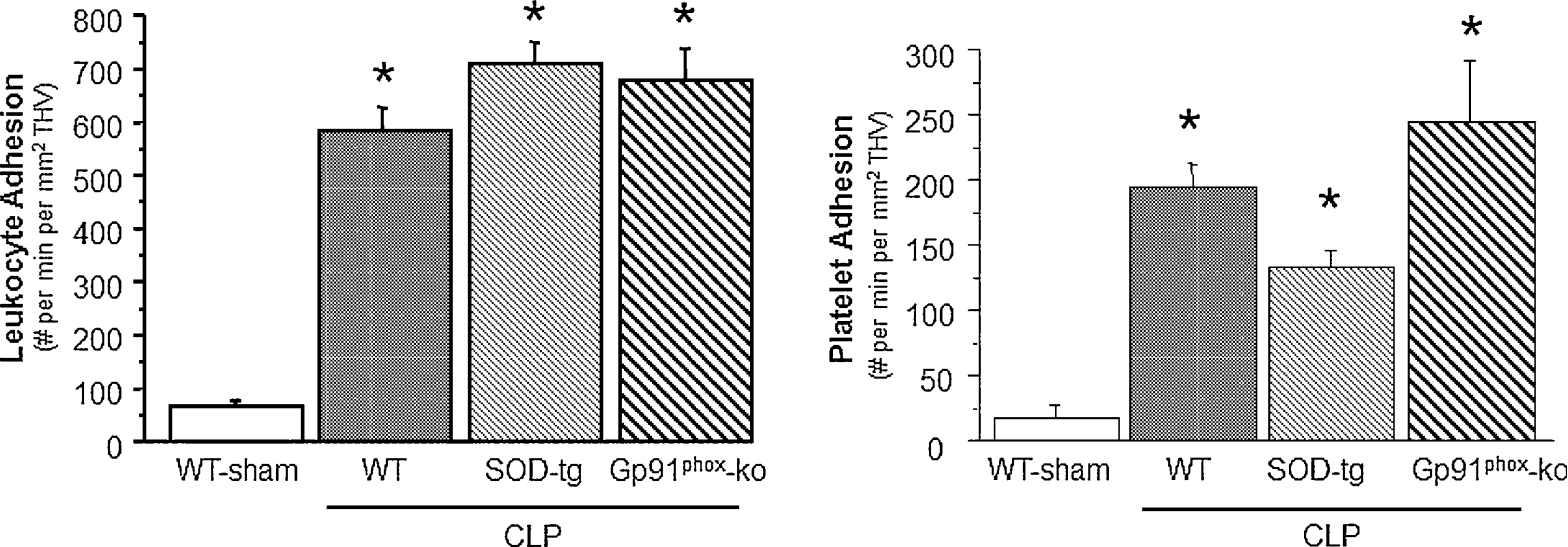
Leukocyte and platelet adhesion in terminal hepatic venules (THV) 6 h following cecal ligation and puncture (CLP, *n* = 8) or a sham procedure in WT (*n* = 8), CLP in SOD-tg (*n* = 8) and gp91phox-ko (*n* = 5) mice. *p < 0.05 versus WT-sham mice. *WT* wild type, *SOD* superoxide dismutase

**Fig. 2:**
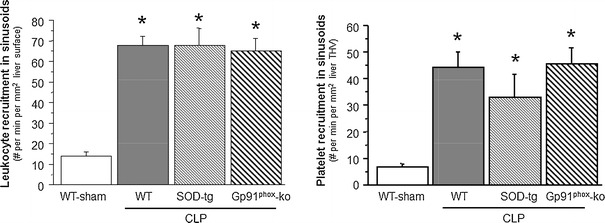
Recruitment of leukocytes and platelets to hepatic sinusoids 6 h following cecal ligation and puncture (CLP, *n* = 8) or a sham procedure in WT (*n* = 8), CLP in SOD-tg (*n* = 8) and gp91phox-ko mice (*n* = 5). *p < 0.05 versus WT-sham mice. *WT* wild type, *SOD* superoxide dismutase

**Fig. 3:**
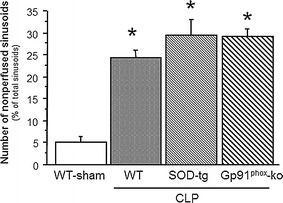
Number of nonperfused sinusoids in WT, SOD-tg (*n* = 8) and gp91phox-ko (*n* = 5) mice 6 h post-CLP (*n* = 8), compared to a WT-sham group (*n* = 8). *p < 0.05 versus WT sham mice. *WT* wild type, *SOD* superoxide dismutase, *CLP* cecal ligation and puncture

Figure 1 summarizes the changes in leukocyte and platelet adhesion in terminal hepatic venules (THV) observed in WT, SOD-tg and gp91^phox^-ko mice 6 h following induction of sepsis using CLP. Compared to the WT sham group, CLP caused significantly elevated adhesion of both leukocytes and platelets in WT, SOD-tg and gp91^phox^-ko mice. A similar pattern of leukocyte and platelet recruitment responses was noted in hepatic sinusoids (Fig. 2) of the tested mouse strains after CLP. The elevated blood cell recruitment in sinusoids was accompanied by a significant reduction in the density of perfused sinusoids. Figure 3 demonstrates an approximately 5-fold increase of the percentage of non-perfused sinusoids in WT, SOD-tg and gp91^phox^-ko mice at 6 h post-CLP when compared to WT-sham, with no differences between the CLP groups.

### Reactive nitrogen species experiments

**Fig. 4:**
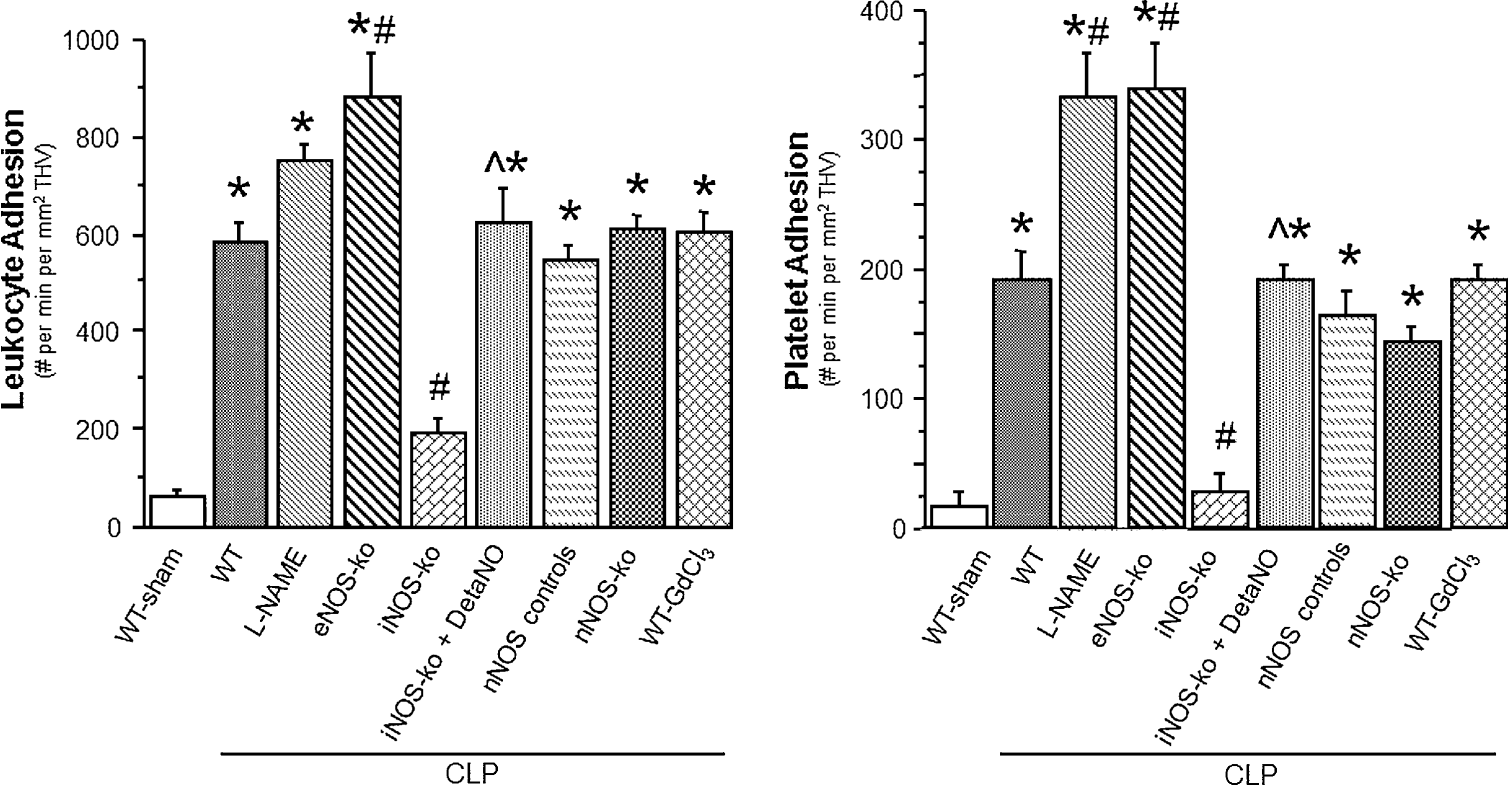
Leukocyte and platelet adhesion in terminal hepatic venules (THV) 6 h following cecal ligation and puncture (CLP) in WT mice (*n* = 8), WT mice treated with l-NAME (*n* = 5), eNOS-ko mice (*n* = 5), iNOS-ko mice (*n* = 5), iNOS-ko mice treated with DetaNO (*n* = 5), nNOS control mice (*n* = 6), nNOS-ko mice (*n* = 5) and WT mice treated with GdCl_3_ (*n* = 5) compared to a group of WT mice following a sham procedure (*n* = 8). *p < 0.05 versus WT sham mice, ^#^ *p* < 0.05 versus WT-CLP, ^^^ *p* < 0.05 versus iNOS-ko-CLP. *WT* wild type, *l* *-NAME* n-nitro-l-arginine methyl ester, *eNOS* endothelial nitric oxide synthase, *iNOS* inducible nitric oxide synthase, *DetaNO* diethylenetriamine nitric oxide, *nNOS* neuronal nitric oxide synthase, *GdCl*_*3*_ gadolinium chloride

**Fig. 5:**
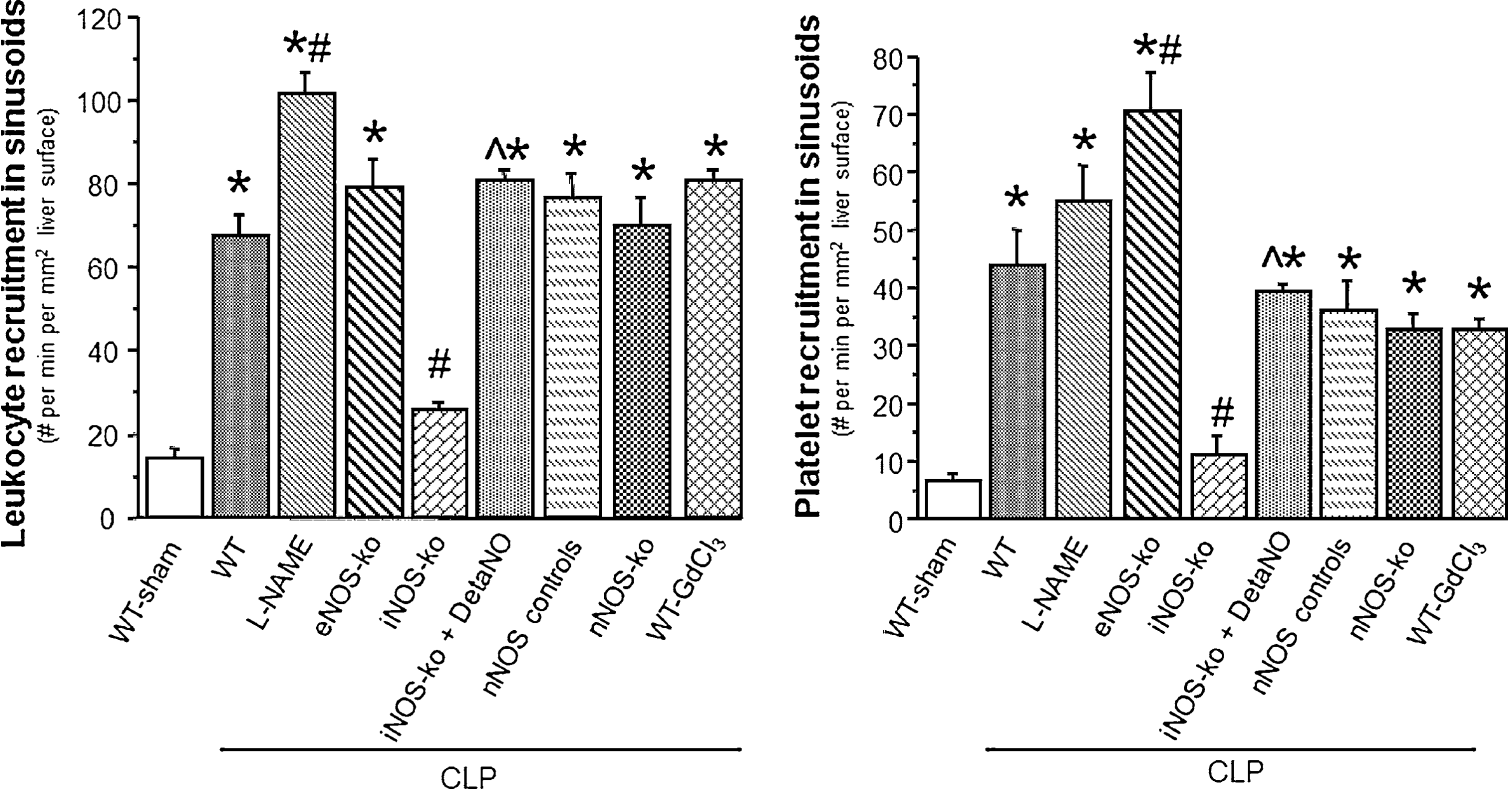
Recruitment of leukocytes and platelets to hepatic sinusoids 6 h following cecal ligation and puncture (CLP) in WT mice (*n* = 8), WT mice treated with l-NAME (*n* = 5), eNOS-ko mice (*n* = 5), iNOS-ko mice (*n* = 5), iNOS-ko mice treated with DetaNO (*n* = 5), nNOS control mice (*n* = 8), nNOS-ko mice (*n* = 5) and WT mice treated with GdCl_3_ (*n* = 5) compared to a group of WT mice following a sham procedure (*n* = 8). *p < 0.05 versus WT sham mice, ^#^ *p* < 0.05 versus WT-CLP, ^^^ *p* < 0.05 versus iNOS-ko-CLP. *WT* wild type, *l* *-NAME* n-nitro-l-arginine methyl ester, *eNOS* endothelial nitric oxide synthase, *iNOS* inducible nitric oxide synthase, *DetaNO* diethylenetriamine nitric oxide, *nNOS* neuronal nitric oxide synthase, *GdCl*_*3*_ gadolinium chloride

**Fig. 6:**
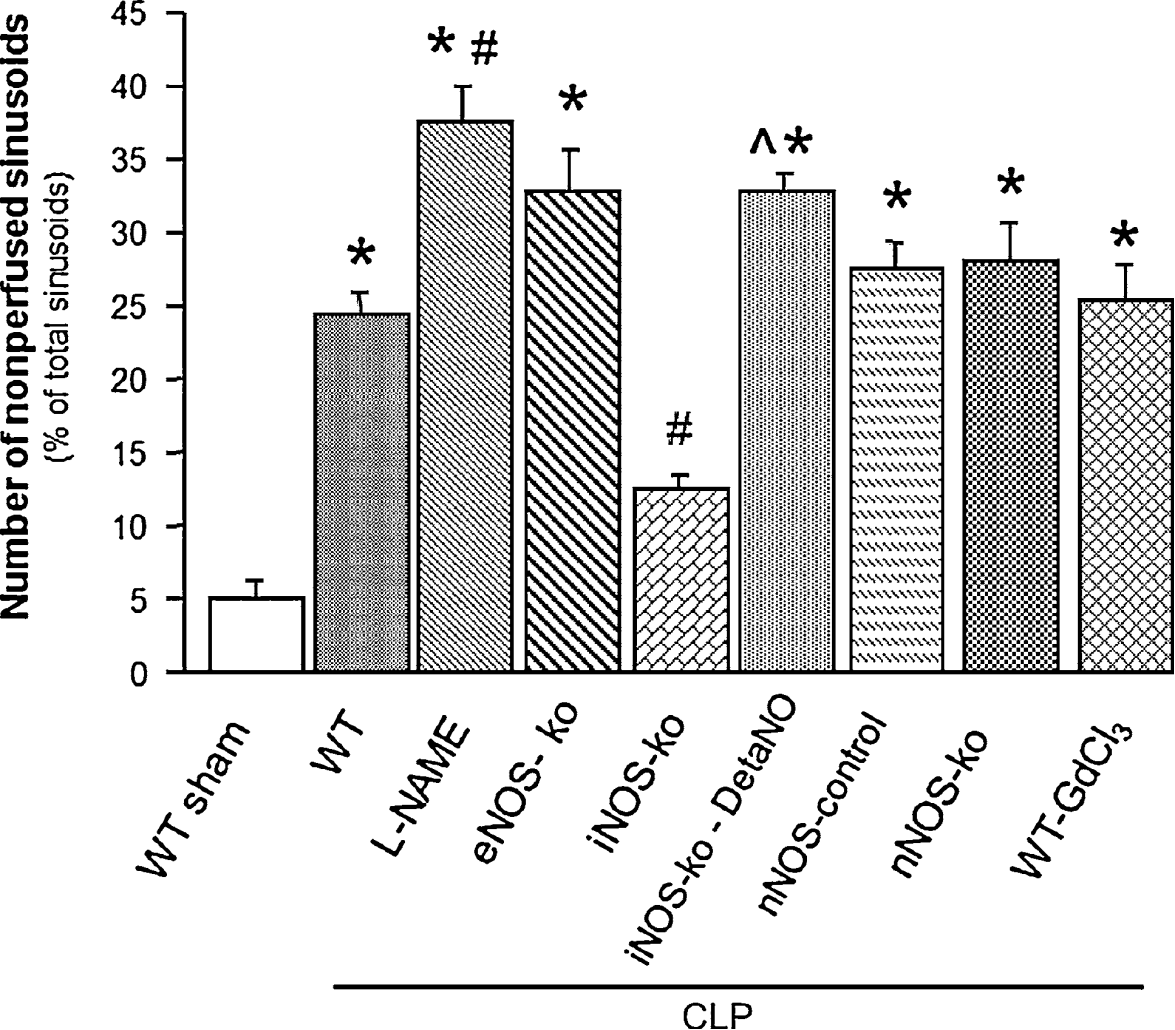
Number of nonperfused sinusoids in WT mice (*n* = 8), WT mice treated with l-NAME (*n* = 5), eNOS-ko mice (*n* = 5), iNOS-ko mice (*n* = 5), iNOS-ko mice treated with DetaNO (*n* = 5), nNOS control mice (*n* = 8), nNOS-ko mice (*n* = 5) and WT mice treated with GdCl_3_ (*n* = 5) compared to a group of WT mice following a sham procedure (*n* = 8). *p < 0.05 versus WT sham mice, ^#^ *p* < 0.05 versus WT-CLP, ^^^ *p* < 0.05 versus iNOS-ko-CLP. *WT* wild type, *l* *-NAME* n-nitro-l-arginine methyl ester, *eNOS* endothelial nitric oxide synthase, *iNOS* inducible nitric oxide synthase, *DetaNO* diethylenetriamine nitric oxide, *nNOS* neuronal nitric oxide synthase, *GdCl*_*3*_ gadolinium chloride

Table 2 depicts the changes in MAP, serum ALT concentration and the blood count of leukocytes and platelets in WT mice, WT mice treated with l-NAME, eNOS-ko, iNOS-ko, iNOS-ko treated with DetaNO, nNOS-ko controls, nNOS-ko and WT mice treated with GdCl_3_ mice following CLP. Although serum-ALT levels did not differ between groups, leukocyte counts were significantly reduced in all mice following CLP. Platelet counts showed a tendency to decline, which reached significance only in WT-CLP and nNOS control mice following CLP. The MAP was significantly elevated in eNOS-ko mice at 6 h after the induction of sepsis. Figure 4 shows both the leukocyte and platelet adhesion in THV. Cecal ligation and puncture lead to a significantly increased adhesion of leukocytes and platelets in WT mice, nNOS control mice and nNOS-ko mice. WT mice treated with l-NAME exhibited an even higher adhesion of platelets to THV, and genetic deficiency of eNOS led to enhanced adhesion of both leukocytes and platelets. While iNOS-ko mice were protected against the CLP-induced leukocyte and platelet adhesion, treatment of iNOS-ko mice with DetaNO restored the pro-adhesive phenotype of CLP. The recruitment of adherent leukocytes and platelets in hepatic sinusoids exhibited a largely comparable pattern, with DetaNO treatment once again restoring the adhesion response (Fig. 5). These blood cell recruitment responses were also accompanied by hepatic sinusoidal perfusion failure (Fig. 6), which was not detected in iNOS-ko mice unless they were treated with DetaNO. Depletion of Kupffer cells using GdCl_3_ did not confer protection against any of the blood cell recruitment or the sinusoidal perfusion failure observed in the liver in response to CLP.

**Table 2:** Mean arterial pressure (MAP), serum ALT concentration, and blood leukocyte and platelet counts in wild type and mutant mice targeting nitric oxide bioavailability

	MAP (mmHg)	ALT (IU/l)	Leukocyte count (#/μl)	Platelet count (# × 10^3^/μl)
WT-sham (*n* = 4)	60 ± 3	45 ± 9	4380 ± 250	1132 ± 33
WT-CLP (*n* = 7)	51 ± 4	53 ± 7	980 ± 150^*^	544 ± 34^*^
WT-l-*n*AME + CLP (*n* = 5)	69 ± 7	75 ± 21	1970 ± 150^*^	803 ± 124
eNOS-ko + CLP (*n* = 6)	88 ± 3^#^	64 ± 9	2160 ± 390^*^	845 ± 79
iNOS-ko + CLP (*n* = 6)	65 ± 3	54 ± 4	2330 ± 320^*^	815 ± 95
iNOS-ko + CLP + DetaNO (*n* = 6)	66 ± 6	42 ± 3	1460 ± 110^*^	694 ± 81
nNOS controls + CLP (*n* = 6)	74 ± 8	55 ± 8	2280 ± 520^*^	625 ± 53^*^
nNOS-ko + CLP (*n* = 6)	67 ± 7	35 ± 3	2470 ± 660^*^	710 ± 42
WT-GdCl_3_-CLP (*n* = 6)	47 ± 7	38 ± 2	2220 ± 440^*^	670 ± 83

Values are mean ± SE

*MAP* mean arterial pressure, *ALT* alanine aminotransferas, *CLP* cecal ligation and puncture, *eNOS* endothelial nitric oxide synthase, *iNOS* inducible nitric oxide synthase, *nNOS* neuronal nitric oxide synthase, *GdCl*_*3*_ gadolinium chloride

* *p* < 0.05 versus WT sham, ^#^ *p* < 0.05 versus WT CLP

### Serum levels of cytokines

**Fig. 7:**
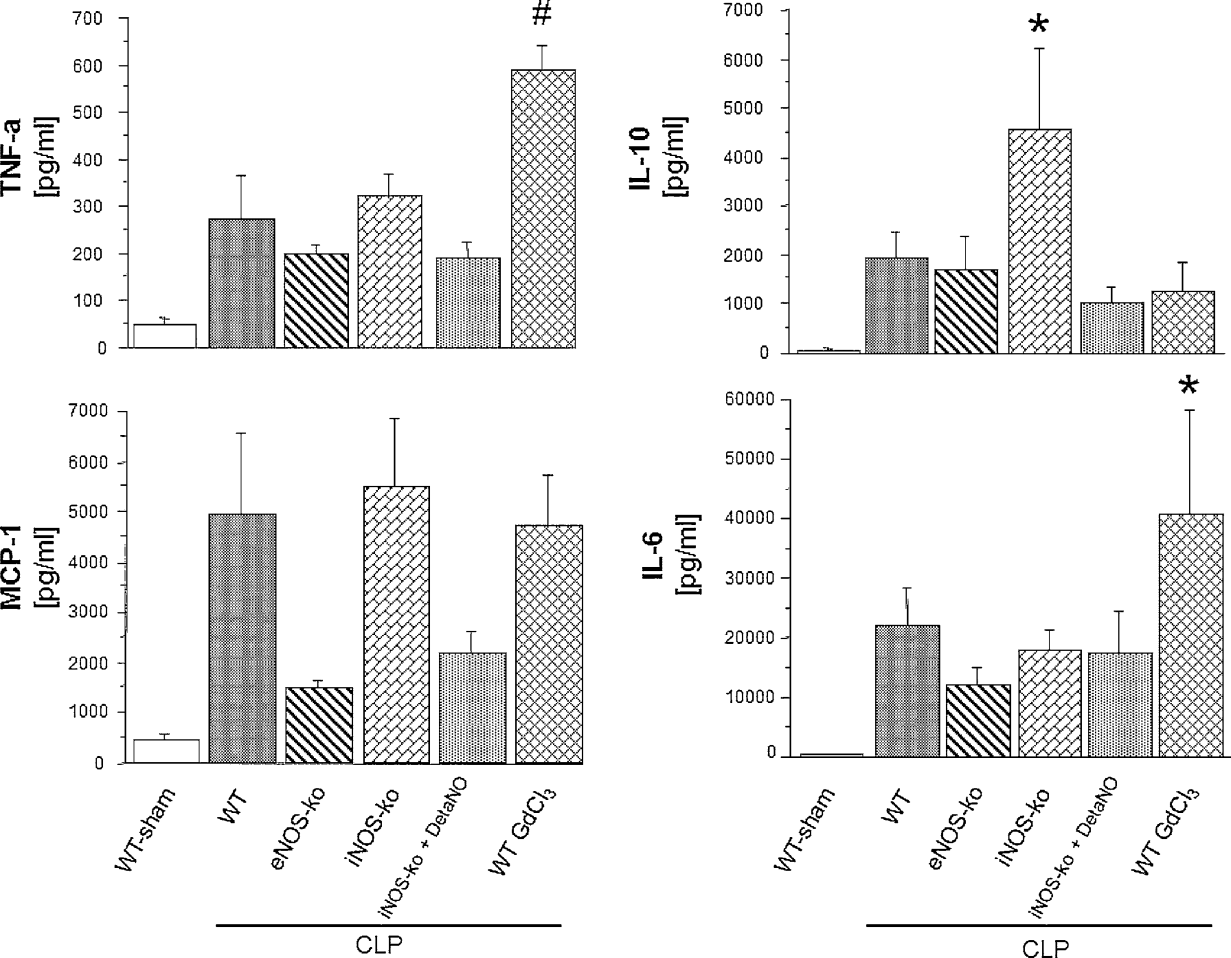
Serum levels of TNF-a, MCP-1, IL-10 and IL-6 six hours following induction of sepsis using cecal ligation and puncture (CLP) in WT mice (*n* = 6), eNOS-ko mice (*n* = 5), iNOS-ko mice (*n* = 5), iNOS-ko mice treated with DetaNO (*n* = 6) and WT mice treated with GdCl_3_ (*n* = 5) compared to a group of WT mice following a sham procedure (*n* = 6). *p < 0.05 versus WT sham mice, ^#^ *p* < 0.05 versus WT-sham and WT-CLP. *TNF-*α tumor necrosis factor alpha, *MCP-1* monocyte chemoattractant protein-1, *IL-10* interleukin 10, *IL-6* interleukine-6, *eNOS* endothelial nitric oxide synthase, *iNOS* inducible nitric oxide synthase, *DetaNO* diethylenetriamine nitric oxide, *GdCl*_*3*_ gadolinium chloride

Figure 7 depicts changes in serum cytokine levels following CLP in WT, eNOS-ko, iNOS-ko, iNOS-ko treated with DetaNO, and WT mice treated with GdCl_3_ compared to WT mice following a sham procedure. TNF-α levels were only elevated in mice treated with GdCl_3_ following CLP. While IL-10 was significantly elevated in iNOS-ko mice, only a tendency for elevated levels of MCP-1 was observed in WT, iNOS-ko and WT mice treated with GdCl_3_ at 6 h post-CLP. IL-6 levels were increased in the GdCl_3_ treated WT mice following induction of sepsis.

In order to confirm that the changes of cytokines in the GdCl_3_ treated mice were not due to the depletion of Kupffer cells only, levels of cytokines were assessed in a group of mice following treatment with GdCl_3_ and a sham procedure. While cytokine levels in mice treated with GdCl_3_ and CLP were elevated to levels comparable to, or above those, in WT CLP mice, mice following GdCl_3_ treatment without CLP exhibited levels of these cytokines that were comparable to the WT sham group (data not shown). Serum levels of both IFN-γ and IL-12 were not altered in any group of mice studied.

## Discussion

Reactive oxygen and nitrogen species have received considerable attention as potential mediators of the tissue injury and organ failure that accompanies sepsis. The liver, with its large population of resident macrophages (Kupffer cells), has the capacity to generate high concentrations of both ROS and NO in response to septic stimuli [21]. In addition, sepsis is associated with the recruitment of leukocytes and platelets into the hepatic microvasculature, which can also contribute to the elevated fluxes of ROS [22] and NO [23]. In the present study, we determined whether NADPH oxidase, superoxide and/or NO, derived from any of the 3 major NOS isoforms, play a role in mediating the leukocyte and platelet recruitment as well as the impaired capillary perfusion that is elicited in the liver microcirculation in the CLP model of sepsis. Overall, our findings support a major role for iNOS-derived NO, with no role for NADPH oxidase derived superoxide, in the hepatic microvascular dysfunction that accompanies CLP-induced sepsis.

It has previously been reported that CLP-induced sepsis elicits a highly significant recruitment of both leukocytes and platelets in the liver microvasculature and this is accompanied by sinusoidal malperfusion [18]. The vast majority of the adherent leukocytes in this model were shown to be polymorphonuclear neutrophils [18], which respond vigorously to septic stimulation by producing ROS and NO [24]. While the respiratory burst is directed to the killing of bacteria, excess quantities of ROS can lead to vascular injury and ultimately organ dysfunction. Superoxide is also known to promote the recruitment of inflammatory cells and platelets into the microcirculation following administration of LPS [25]. Furthermore, superoxide has been implicated in the sinusoidal malperfusion that is associated with ischemia/reperfusion in mouse liver [26]. NADPH oxidase has been shown to be a major source of the superoxide generation that is associated with sepsis in the lung [11]. Gao and coworkers demonstrated an enhanced sequestration of neutrophils, but a reduction in microvascular permeability, in the lungs of p47^phox^ and gp91^phox^ deficient mice challenged i.p. with live E. coli, compared to their WT counterparts. In contrast, the results of the present study showed no effects of gp91^phox^ deficiency on the liver microvascular and inflammatory responses to sepsis, suggesting that NADPH oxidase is not a major participant in the hepatic microvascular responses to polymicrobial sepsis elicited by CLP.

It is commonly believed that NO produced by nNOS and eNOS mediates important physiologic processes (e.g., vasodilation) while iNOS-derived NO mediates pathological responses in conditions like sepsis [27]. Nonetheless, both protective and deleterious effects of eNOS-derived NO in sepsis have been reported [28, 29]. Unfortunately, the conclusions drawn in most of these studies were largely based on the use of nonselective inhibitors of eNOS. Our own findings indicate that mice that are genetically deficient in eNOS and mice treated with the NOS inhibitor L-NAME tend to exhibit an exacerbated blood cell recruitment response in the hepatic microvasculature following CLP, suggesting that eNOS-derived NO normally exerts a protective effect in preventing an accumulation of platelets and leukocytes. This notion is supported by Eum and coworkers who demonstrated that L-NAME treatment augmented CLP-induced elevation of liver enzymes [30]. The other constitutive isoform of NOS, nNOS, does not appear to play a role in the liver response to sepsis as reflected by the data obtained from nNOS deficient mice.

The responses to the high levels of NO generated by iNOS can be variable, with several studies demonstrating a role for the inducible isoform of NOS, iNOS, in the pathogenesis of sepsis, whereas others have failed to show a beneficial effect of iNOS inhibition on septic organ damage [31, 32]. By utilizing iNOS selective inhibitors or iNOS deficient mice, it has been shown that the NO derived from this enzyme mediates some of the deleterious responses to sepsis in organ systems such as the cremasteric muscle [33]. The results of the present study demonstrate a dramatic reduction of blood cell recruitment and improved sinusoidal perfusion in iNOS deficient mice subjected to CLP, which supports the view that iNOS derived NO is an important contributor to these responses. Our findings are supported by reports describing a substantially reduced upregulation of E- and P-selectin in iNOS deficient mice following CLP in several other organs [34]. The importance of NO in mediating the responses observed in the iNOS knockout mice is illustrated by our ability to reverse the “protection” afforded by iNOS deficiency by administering a NO donating agent (DetaNO) to these mutant mice.

Six hours following induction of sepsis, we were not able to demonstrate the hypotension which is a hallmark of sepsis and septic shock. This is consistent with reports showing that—after CLP—hypotension develops at a later time point [35]. The progressive hypotension reflects the profile characteristic of clinical sepsis [36]. However, in mice lacking eNOS hypertension is commonly described, and reflects the findings in our experiments [37].

Another noteworthy observation in the present study was the absence of protection afforded by depletion of Kupffer cells using gadolinium chloride. Using the same GdCl_3_ treatment regimen in a murine model of ischemia–reperfusion (I/R)-induced liver inflammation, we demonstrated that Kupffer cell depletion significantly blunts the leukostasis and sinusoidal malperfusion induced by I/R [38]. Given the absence of protection in the setting of CLP and the protection afforded by iNOS deficiency, our findings would also suggest that Kupffer cells are an unlikely source of the iNOS that mediates the microvascular responses to sepsis in our model [39]. Other possible sources of iNOS in the liver include hepatocytes as well as leukocytes [40, 41]. The lack of protection from the hepatic microvascular disturbances in septic mice following depletion of Kupffer cells may be related to the increased levels of TNF-α and IL-6. Our findings are consistent with a report showing elevated levels of TNF-α in Kupffer cell depleted mice following a septic stimulus [42].

Genetic ablation of iNOS has been shown to modulate cytokine levels ([43]. One interesting finding in our study was the markedly increased values of IL-10 in iNOS deficient mice following CLP. The anti-inflammatory effects of IL-10 may be related to the decreased hepatic microvascular disturbances following sepsis in iNOS knockout mice.

The results of the present study show that the hepatic microcirculatory failure associated with CLP does not seem to be mediated by NADPH oxidase. In contrast, our findings clearly support the view that iNOS is a relevant therapeutic target for protection of the liver dysfunction that is known to accompany human sepsis. However, considering the contradictory results of studies highlighting the pathophysiological role of iNOS during sepsis, more research has to be done in order to shed light on the mechanisms that underlie the deleterious effects of iNOS-derived NO during sepsis.
